# Complex PTSD, affect dysregulation, and borderline personality disorder

**DOI:** 10.1186/2051-6673-1-9

**Published:** 2014-07-09

**Authors:** Julian D Ford, Christine A Courtois

**Affiliations:** University of Connecticut Health Center MC1410, 263 Farmington Avenue, Farmington, CT 06030-1410 USA; Independent Pactice, Washington, DC, Elements Behavioral Health, Promises, Malibu, CA USA

**Keywords:** Borderline personality disorder, Complex PTSD, Dissociation, Affect dysregulation

## Abstract

Complex PTSD (cPTSD) was formulated to include, in addition to the core PTSD symptoms, dysregulation in three psychobiological areas: (1) emotion processing, (2) self-organization (including bodily integrity), and (3) relational security. The overlap of diagnostic criteria for cPTSD and borderline personality disorder (BPD) raises questions about the scientific integrity and clinical utility of the cPTSD construct/diagnosis, as well as opportunities to achieve an increasingly nuanced understanding of the role of psychological trauma in BPD. We review clinical and scientific findings regarding comorbidity, clinical phenomenology and neurobiology of BPD, PTSD, and cPTSD, and the role of traumatic victimization (in general and specific to primary caregivers), dissociation, and affect dysregulation. Findings suggest that BPD may involve heterogeneity related to psychological trauma that includes, but extends beyond, comorbidity with PTSD and potentially involves childhood victimization-related dissociation and affect dysregulation consistent with cPTSD. Although BPD and cPTSD overlap substantially, it is unwarranted to conceptualize cPTSD either as a replacement for BPD, or simply as a sub-type of BPD. We conclude with implications for clinical practice and scientific research based on a better differentiated view of cPTSD, BPD and PTSD.

## Review

Individuals who experience complex (i.e., developmentally adverse interpersonal) trauma (e.g., maltreatment, prolonged family or community violence, torture or exploitation) —especially but not exclusively in formative periods (e.g., early childhood, adolescence)—are at risk not only for posttraumatic stress disorder (PTSD) but also other psychiatric disorders [1], including personality disorders [2–5]. The role of traumatic stress in the etiology and phenomenology of Borderline Personality Disorder (BPD) has received substantial attention [6] because BPD poses particularly difficult clinical challenges consistent with the developmental aberrations associated with developmental trauma [7–9].

Two decades ago, complex PTSD (cPTSD) was defined as a syndrome involving pathological dissociation, emotion dysregulation, somatization, and altered core schemas about the self, relationships, and sustaining beliefs (i.e., morality, spirituality) in the aftermath of exposure to traumatic interpersonal victitmization. cPTSD was proposed as an alternative for conceptualizing and treating the symptoms of adults who had suffered prolonged and severe interpersonal trauma, many of whom were diagnosed with BPD [10]. Since then, hundreds of clinical or scientific studies of cPTSD and cognates (e.g., Disorders of Extreme Stress [11, 12]; Developmental Trauma Disorder [13, 14] have been published. However, the relationship of cPTSD to BPD remains in question, and this question is the focus of the present review.

cPTSD has been both proposed and disputed as a valid and useful [15] clinical syndrome [16, 17]. Questions include whether cPTSD is simply a re-packaging or duplication of BPD or whether it provides a rational basis for enhancing the precision of assessment and diagnosis and the range of treatment options for individuals whose BPD symptoms and impairment are caused or exacerbated by the complex traumatic stress reactions [18]. A principal difference between cPTSD and BPD is the assumption in the core definition of cPTSD that its symptoms are sequelae of exposure to traumatic stress, which is not inherent in the definition of BPD. To address these issues, we will review clinical and scientific findings regarding: (1) comorbidity and clinical phenomenology of PTSD, cPTSD and BPD; (2) the role of traumatic victimization in BPD and the possibility that cPTSD could represent a sub-type of BPD; (3) the psychobiology of PTSD, cPTSD, and BPD; and, (4) whether a cPTSD conceptualization could clarify the relationship of two hypothesized core mechanisms in BPD, dissociation and affect dysregulation. We conclude with implications for clinical practice and scientific research based on a better differentiated view of PTSD, cPTSD, and BPD.

### Prevalence and comorbidity of BPD, PTSD, and cPTSD

The estimated prevalence in the adult population in the United States of BPD is 6% [19]. By comparison, across a range of post-industrial nations, the estimated lifetime prevalence of PTSD in adults ranged from 1-10% [20, 21]. cPTSD prevalence estimates have not been reported in community or primary healthcare samples.

PTSD is often noted as a common comorbidity of BPD [22–25]. In nationally representative samples in the United States, approximately 30% of adults meeting criteria for either PTSD or BPD also met criteria for the other disorder, and closer to 40% of adults diagnosed with BPD had an episode of PTSD at some point in their lifetime [19]. A 10-year follow-up of adults diagnosed with BPD found that most (85%) who initially were diagnosed with PTSD continued to meet criteria for BPD but experienced a remission of PTSD [26, 27]. However, almost half of remitted PTSD cases experienced a recurrence. Individuals with childhood histories of sexual abuse were least likely to remit on PTSD. More than one in four of the BPD cohort had a new diagnosis of PTSD over the 10-year period, most often following a sexual assault [26, 27]. These findings closely parallel those for remission, recurrence, and new cases of substance use disorders (SUD) [26, 27], suggesting a need for examination of the course and risk factors for complex comorbid combinations of PTSD, SUD, and BPD. They also speak to the risk of revictimization that has been found to be associated with complex traumatization (especially sexual abuse) in childhood [28, 29].

Despite not yet being formally accepted as a psychiatric diagnosis in the *Diagnostic and Statistical Manual (IV-TR and 5)* (American Psychiatric Association, 2000; 2013), cPTSD also has been reported to frequently co-occur with BPD in both outpatient and inpatient psychiatric and SUD treatment populations [30–36]. As a proxy for cPTSD, several studies have shown that the combination of a history of childhood victimization and *DSM-*IV PTSD are associated with distinct BPD presentations, including: (1) deficits in cognitive empathy [37] and cognitive affect management [38], (2) suicide attempts [22, 39] and lethality [40], (3) non-suicidal self-injury [40, 41], (4) crises leading to hospitalization [25], (5) psychotic symptoms [42], (6) anxious and guilt-prone (rather than disgust- or shame-based) self- concept [43–45], and (7) in approximately 30% of a sample of patients diagnosed with BPD, obesity [46]. In addition, individuals with a BPD diagnosis or symptoms have been shown to have distinct psychophysiological profiles when PTSD is a comorbidity, including; (1) structural brain abnormalities (i.e., increased gray matter volume) prefrontal cortex areas associated with cognitive control [47], (2) altered brain function (i.e., increased insula and decreased parahippocampal activation in association with increasingly severe dissociation symptoms) [48], (3) reduced amygdala volume [38, 49] and altered amygdala metabolism [50], (4) reduced hippocampus volume and increased impulsivity [49], and, (5) neuroendocrine stress hyporeactivity (i.e., cortisol suppression) [51–53].

However, *DSM-*IV PTSD and child abuse history also have been found to be unrelated to BPD symptomatic or impairment severity [25, 41, 45, 54–58]. Thus, while PTSD may complicate BPD, a more precise formulation of the specific types of trauma-related dysregulation hypothesized to be involved in cPTSD may be necessary in order to account for the role of trauma in BPD.

### Clinical phenomenology of BPD, PTSD, and cPTSD

BPD and PTSD are relatively distinct with regard to the precise qualitative definitions of their diagnostic features, but nevertheless have substantial potential overlap in their symptom criteria. In the *DSM*-IV (American Psychiatric Association, 2000) and ICD-10 [10], PTSD symptoms (i.e., dissociative amnesia and flashbacks; emotional numbing; anger) were similar to three BPD features (i.e., transient dissociation, chronic emptiness, and intense anger, respectively). Moreover, the *DSM*-5′s (American Psychiatric Association, 2013) revised PTSD criteria include new symptoms reflecting pervasive negative changes in affect, identity, and behavior which overlap with four other BPD criteria (i.e., identity disturbance, potentially self-damaging impulsivity, self-harm, affective instability). The *DSM*-5 PTSD diagnostic criteria thus potentially overlap with seven of nine BPD criteria.

The new *DSM-*5 PTSD symptoms are notable for their relationship to cPTSD. In fact, all of the new or revised PTSD symptoms that potentially overlap with BPD features are cardinal features of cPTSD: affect dysregulation, altered core beliefs about self, reckless or dangerous impulsive behavior, self-harm. In addition, cPTSD involves intense and volatile enmeshment in relationships as a core feature as well as the social detachment and avoidance that are *DSM-IV* and *DSM-*5 PTSD criteria [59]:

- Proposed ICD-11 complex PTSD is a disorder that requires PTSD symptoms … but also includes three additional features that reflect the impact that trauma can have on systems of self-organization, specifically problems in affective, self-concept, and relational domains. … The affective domain problems are characterized by emotion dysregulation as evidenced by heightened emotional reactivity, violent outbursts, reckless or self-destructive behavior, or a tendency towards experiencing prolonged dissociative states when under stress. In addition, there may be emotional numbing and a lack of ability to experience pleasure or positive emotions. Self-disturbances are characterized by negative self-concept marked by persistent beliefs about oneself as diminished, defeated or worthless [possibly] accompanied by deep and pervasive feelings of shame or guilt… Interpersonal disturbances are defined by persistent difficulties in sustaining relationships [due to a tendency to either] avoid, deride or have little interest in relationships [or] occasionally experience[ing] close or intense relationships but [having] difficulty maintaining emotional engagement.

The core features of dysphoria in BPD—perceived betrayal and victimization, fragmented sense of self and desire to self-harm, and perceived inability to control extreme affects and impulses [24, 60, 61]—closely mirror the cPTSD features of relational, self, and affect dysregulation [59], and cPTSD’s association with traumatic betrayal [62] and the persistent belief that trauma has created a permanently damaged self and related disorders of the self [10].

However, two BPD diagnostic criteria specifically related to attachment disorganization or insecurity [63] are quite distinct from PTSD in the *DSM-*5 and from cPTSD: terror of abandonment or rejection, and alternating idealization and devaluation of others. This is consistent with the laboratory research finding that physiological reactivity by individuals with childhood sexual or physical abuse histories who met criteria for BPD was strongest when exposed to scripts highlighting themes of abandonment, while those meeting criteria for PTSD but not BPD had peak physiological reactivity when exposed to scripts of traumatic (e.g., violent, abusive, life-threatening) events [64].

### The role of trauma exposure in BPD

Adults diagnosed with BPD have been shown to be more likely than adults with other psychiatric or personality disorders or no psychopathology to report a history of psychological trauma [5, 65–70]. Interpersonal victimization in childhood has been found to be highly prevalent among adults with BPD [71–73] and adolescents with BPD features [74, 75]. For example, in a sample of adults in psychiatric treatment diagnosed with BPD, 81% reported histories of interpersonal trauma in childhood, including physical abuse (71%), sexual abuse (68%), and witnessing domestic violence (62%) [76].

Adults with BPD also have been found to be more likely than psychiatric or non-clinical controls to report childhood emotional abuse [77] and neglect or impaired caregivers [67, 69, 78, 79]. As a result, traumatic victimization and compromised primary caregiving relationship have been hypothesized to be key etiological contributors to BPD [7, 80, 81].

Adults with BPD also are at risk for abuse or re-victimization in adulthood [66, 67, 82], and cumulative poly-victimization across the lifespan [39, 66, 67, 70, 82]. Although severe childhood sexual abuse (i.e., prolonged, violent, multiple perpetrators, physical penetration) was found to be the childhood trauma type most consistently associated with BPD symptoms and impairment [70], neglect likely contributes to, or is a risk factor for BPD [83] because sexual abuse often co-occurs with neglect —and neglect has been shown to be a separate risk factor for BPD [69].

Moreover, BPD has a multi-factorial etiology, and trauma is only one of many potential risks or contributors. Genetics (e.g., temperament) and formative developmental experiences (e.g., maltreatment, caregiving, extra-familial relationships) likely interact in complex ways in BPD etiology [71, 80, 81, 84–87]. The sense of profound emotional pain that was described as “the essential nature” of BPD [88] could be due to traumatic victimization in childhood or adulthood, but could alternately develop in the absence of trauma or victimization when a child experiences disruptions in formative relationships with primary caregiver(s) that lead either actual abandonment or rejection (e.g., neglect; impaired/emotionally unavailable primary caregiver) or perceived abandonment or rejection (e.g., poor fit of child and caregiver due to incompatible temperaments or personalities) [89, 90].

Further, BPD is only one of many psychiatric sequelae for which childhood trauma exposure has been demonstrated to be a risk factor. Independent of BPD, childhood trauma exposure places children and adults at risk for mood (e.g., bipolar or major depressive disorder), anxiety (e.g., panic, social phobia), obsessive-compulsive, eating, dissociative, addictive, psychotic, somatoform, and personality disorders, as well as significant safety problems including suicidality, self-harm, impulsivity, aggression, reckless behavior, self-medication, and re-victimization [1, 72]. When these disorders and severe impairments occur as comorbidities or features of BPD [19, 24, 60, 91–93], these complex cases tend to be particularly chronic, dangerous, and treatment-refractory [8, 9, 94–100]. Trauma exposure also is highly likely in BPD cases with multiple severe comorbidities, with childhood trauma history prevalence estimates exceeding 90% [101–103]. Extreme BPD-plus-comorbidity cases thus may constitute a cPTSD sub-type of BPD, although whether childhood victimization contributes uniquely to these cases remains to be investigated systematically.

However, it is unlikely that cPTSD occurs only as a BPD sub-type. Although comorbidity frequently occurs in BPD cases, BPD is a relatively uncommon comorbidity of other psychiatric disorders. Most cases of Axis I and II disorders do not have comorbid BPD: the highest BPD comorbidity rates are 50% for eating disorders [24], 30-35% for SUD or bipolar disorder, and 25-50% for paranoid, avoidant, dependent, and schizotypal personality disorders. BPD comorbidity is typically found in less than 10% and at most 15-20% of Axis I or II disorders [19]. Similarly, cPTSD often is accompanied by multiple psychiatric comorbidities, but occurs only rarely as a comorbidity in samples of adults with severe mental illness [32, 104], SUD [33], and Axis I disorders including PTSD [11, 12, 31, 105]. In cases where either BPD or cPTSD is a comorbidity of another psychiatric disorders, it is likely that there is a history of the types of intra-familial childhood adversity [1, 5, 79] that have been most consistently found to confer severe impairment related to psychiatric morbidity: “parental mental illness, substance disorder, criminality, family violence, abuse, neglect” [106]. Thus, cPTSD may serve as a sub-type of BPD in which profound developmental trauma causes, or creates a vulnerability to, a range of symptoms and impairments that, in the *DSM*, results in multiple comorbid diagnoses.

Moreover, severe intra-familial early childhood adversity is a well-established risk factor not just for BPD but for the onset of a wide range of adolescent and adult psychiatric disorders [106, 107]. Consistent with a cPTSD perspective, across all psychiatric disorders, cases with the heaviest childhood trauma burden also have the most severe psychosocial impairment [106]. Prevalence estimates of trauma exposure for most psychiatric disorders are similar to those cited above for BPD: SUDs (67-90%) [108], bipolar disorder (50-66%) [109, 110], and other personality disorders (50-78%) [102, 111]. BPD comorbidity with these disorders is much less prevalent than these estimates of trauma exposure prevalence [19]. If cPTSD was primarily or only a sub-type of BPD, then BPD should be present in all high trauma burden Axis I and II disorders—but this is not the case. Similarly, while psychotic symptoms occur in a substantial subset (i.e., 20-50%) of BPD cases [42], psychotic disorders rarely (1%) co-occur with BPD [24]—yet psychotic disorder cases often (50-67%) involve a history of interpersonal trauma [101, 112].

Thus, cPTSD warrants investigation as a potential sub-type of multiple psychiatric disorders, including cases where those disorders do, and do not, have BPD as a comorbidity.

Additionally, there is evidence that childhood trauma exposure and PTSD actually may be more strongly associated with *other* personality disorder diagnoses or features than with BPD. In adult psychiatric samples, childhood trauma history was more strongly related to paranoid personality disorder than BPD [2] and both PTSD and dissociative disorders have been found to be equally or more often comorbid with avoidant, self-defeating, and passive-aggressive personality disorders as with BPD [113]. An epidemiological study identified two sub-groups representing 25% of the PTSD cases, one with BPD features (11.4%) and another with paranoid/obsessive features (13.1%) [114]. The PTSD sub-group with BPD features was more likely than the other PTSD cases to be drug-seeking due to impaired distress tolerance, consistent with evidence of BPD-PTSD-SUD comorbidity [19]. However, it was the paranoid/obsessive sub-type that was most likely to report a history of sexual trauma, and they also had the earliest onset of PTSD. The paranoid and obsessional styles share a common preoccupation with hypervigilance to perceived threat which is consistent with the PTSD’s anxiety and guilt-proneness. That proclivity toward anxiety and guilt also has been shown to be the case when PTSD occurs comorbidly with BPD [43, 44], but differs from the self-hatred [115], shame , prolonged and chronic anger and negative affect associated with BPD *per se.*

Thus, cPTSD appears to involve hypervigilance related to being harmed, while, as noted above, BPD involves extreme sensitivity (which may take the form of hypervigilance) to perceiving oneself as being abandoned or rejected/shamed [63].

### Can BPD be distinguished psychobiologically from PTSD and cPTSD?

As noted above, numerous diagnostic studies have identified a comorbid BPD-PTSD sub-group that differs from BPD alone, both clinically and neurobiologically. BPD has been shown to be associated with smaller hippocampi [38], but a more consistent finding is that reduced hippocampal volume in BPD tends be specific to persons with BPD and comorbid PTSD or childhood maltreatment rather than BPD alone [116]. BPD also is related to reduced amygdala volume—except with comorbid depression [38] and ACC volume [116].

In addition, both BPD diagnosis and childhood sexual abuse history were found to be related to a structural marker for impaired neural network integration (i.e., reduced corpus callosum volume) in a study of women with comorbid BPD and ADHD [43, 44], specifically in pathways connecting the right and left hemisphere loci for emotion regulation and self-monitoring, i.e., the ACC [117]. BPD and avoidant personality disorder patients showed dorsal ACC hypo-activation after viewing repeated emotionally distressing pictures, as well as smaller increases in insula-amygdala functional connectivity and a lack of habituation in their ratings of the emotional intensity of the images [118]. In BPD this failure of biological and psychological emotion habituation was associated with greater affective instability and reduced insula-ventral ACC functional connectivity. BPD also found to be related to ACC and orbital PfC hypo-activation and increased insula and amygdala activation during attempts to distance from distressing emotions [116]. Similarly, ACC hypo-activation occurred when individuals diagnosed with BPD underwent a physical pain induction, although they showed decreased rather than increased amygdala and insula activation, increased dorsolateral PfC activation, and—of note—dissociative analgesia [116]. Amygdala hypo-activation was most pronounced when BPD and PTSD were comorbid, consistent with the dissociative subtype of PTSD [119].

Individuals with BPD and intermittent explosive disorder also showed dorsolateral PfC hypo-activation and elevated orbital PfC and amygdala activation and connectivity in response laboratory tasks eliciting interpersonal frustration [120] and fear [121], similar to the failure of PfC inhibition of amygdala activation found in PTSD and with adults who have child maltreatment histories [122–133]. Whether these functional brain activation patterns are specific to BPD or extend to BPD with childhood interpersonal trauma histories or comorbid PTSD remains to be determined. In BPD, deficits in interpersonal trust, tolerance of aloneness, and recognition of conventional norms of social cooperation/fairness have been documented and shown to be associated with altered patterns of ACC, temporal lobe, and insula activation [121, 134]. These specific forms of interpersonal dysregulation may be distinct to BPD, but whether they are moderated by either childhood maltreatment and PTSD remains to be determined [121]. Thus, unlike the biologically-based emotion dysregulation characterizing fear-related syndromes (e.g., PTSD; avoidant PD), BPD may involve altered brain connectivity associated with emotional instability in response to actual or perceived interpersonal rejection.

The structural and functional brain correlates of cPTSD are less well studied, but there is evidence that individuals with childhood maltreatment histories and cPTSD have reduced hippocampus, ACC, and orbital PfC volumes, with specific correlates of reduced volume in different brain loci: for the ACC, severity of child abuse, PTSD hyperarousal, and BPD; but for the orbital PfC, impulsivity, anger, and BPD [135]. Similar to BPD, cPTSD is associated with a hippocampally-mediated bias toward memory encoding of negative (vs. positive) information [136], but also (in an emotional memory task ) heightened PfC, ACC, amygdala, and hippocampus activation which is the opposite of the diminished PfC inhibition found in studies with either PTSD or BPD [137]. Bryant (personal communication) found similar amygdala and PfC hyper-activation in cPTSD individuals in resting state neuroimaging. These findings suggest that while fear in PTSD and rejection-related distress in BPD may be accompanied by deficient PfC inhibitory activation, in cPTSD they may involve increased—but failed—inhibitory attempts by the PfC.

Physical pain has been found to be problematic in both BPD [88, 138] and PTSD (particularly when the latter is comorbid with depression) [139–141], and to potentially be neurobiologically associated with suicide risk, self-injury, and SUD [142, 143]. However, BPD and PTSD appear to have distinct pain profiles [144]. BPD often involves pain analgesia consistent with dissociation [7] and self-medication with analgesic medications [94], potentially mediated by a neurobiological substrate (i.e., 2-AG endocannabinoids) specific to BPD (versus PTSD) has been identified [145]. In BPD, physical pain is associated with a complex pattern of heightened co-activation of sensory (e.g., basal ganglia), affective (e.g., amygdala), self-referential (i.e., default mode network [DMN], e.g., precuneus, posterior cinglulate), and executive/inhibitory (e.g., medial and dorsolateral PfC) brain areas [146], as well as a less integration and connectivity of the PfC with the DMN and an attenuated DMN response [147] associated with dissociation [48].

Interestingly, adult- or childhood-related PTSD also is associated with heightened synchronization of the fear/salience brain network (centering on the amygdala) and the self-referential DMN (centering on the precuneus and posterior cingulate), and deficient activation of the self-referential DMN [130, 131, 148] and connectivity between the self-referential DMN and executive networks [149]. However, in PTSD, pain-related impairment, hyperalgesia rather than analgesia, is prominent. PTSD secondary to military combat trauma has been shown to be associated with impairment due to physical pain, particularly among veterans with highly negative self-perceptions [150] and depressive symptoms and illness-related pain coping [140]. PTSD associated with childhood abuse in civilian primary care patients also has been found to involve physical pain impairment (and to mediate that relationship), as well as elevated pain severity and problems with emotion dysregulation [151]. The neurobiology of pain in PTSD is not well understood, although PTSD and chronic physical pain appear to be mutually exacerbating in a bidirectional manner [152], yet PTSD also has been found to be associated with reduced sensitivity to externally induced acute pain [153]. Neuroimaging studies have shown that military veterans with PTSD exhibit increased activation in the hippocampus, insula, and putamen, and decreased PfC and amygdala activation in reaction to externally-induced acute pain [153], while women with PTSD secondary to interpersonal violence showed increased activity in both the insula and PfC during acute pain but reduced insula activation with repeated pain induction [154]. When physical pain was induced following script-based recall of traumatic events, PTSD symptom severity was found to be associated with activation in brain areas related to stress-induced analgesia (e.g., caudate, thalamus), and dissociative symptoms were correlated inversely with activation of the putamen and amygdala [155]. Thus, the neurobiology of pain in PTSD is complicated and appears to range from acute analgesia (associated with dissociation) to chronic hyperalgesia (associated with affective distress and hyperarousal).

While preliminary and not directly testing associations with cPTSD, taken together these findings suggest that emotion dysregulation in BPD may involve brain alterations associated with deficient self-awareness, intolerance of interpersonal rejection or abandonment, inability to recover from intense negative affect states, and dissociative analgesia. PTSD secondary to childhood maltreatment, by contrast, appears to involve brain alterations associated with heightened self-awareness of vulnerability, hypervigilance to a wide array of safety threats and related threat appraisals, tolerance of (and possibly habituation to) chronic negative affect states, and dissociative re-experiencing with alternating states of fear/hyperalgesia and detachment/analgesia. PTSD uniformly also involves persistent fear manifested as physiological *hyper*arousal due to an explicit or implicit attentional bias toward (i.e., hypervigilance), or avoidance of, actual or perceived threats [59, 156].

In the *ICD*-10 and *DSM*-IV, *hypo*arousal also is included in PTSD in the form of dissociative amnesia, emotional numbing, and anhedonia, and the *DSM*-5 a hypoarousal-based dissociative sub-type [119]. cPTSD adds to the core PTSD fear symptoms three forms of dysregulation which may involve either or both hyperarousal and hypoarousal: (1) emotion processing, (2) self-organization (including bodily integrity), and (3) relational engagement [33, 59, 156, 157]).

A common denominator across the newer PTSD features also is central to BPD: under-regulation of extreme negative affect states [35, 36, 158, 159]. In both BPD and PTSD secondary to childhood maltreatment, under-regulation of affect are marked by inability to recover from episodes of chronic intense negative affect [75, 121] and dissociation [7]. Therefore, investigation of how dissociation and affect dysregulation in BPD and cPTSD have shared or different patterns of brain structure and function, and associated clinical phenomenology, is crucial to determining the nosological and scientific relationship of BPD and cPTSD.

BPD has been shown to involve deficits in social cognition related to dichotomous thinking, distrust, aggressive attributions, and increased attention to, but impaired recognition, understanding, and empathy for, others’ emotions, thoughts, and intentions. Alterations in brain structure (e.g., cortical thickness) and activation (in the prefrontal and temporal cortices) associated with BPD social cognitive deficits also have been identified [37, 160–166]. Research on social cognition in PTSD is more limited, but one study found that women with BPD were more likely to have empathic deficits if they had comorbid PTSD with severe intrusive re-experiencing and a history of sexual trauma [165]. PTSD by definition involves a numbing of emotion awareness and detachment from interpersonal relationships secondary to an attention bias toward threat [167, 168], consistent with evidence that PTSD may have a dampening effect on emotional reactivity in BPD [51, 52]. Further study is needed both of the differences in altered social cognition in BPD and PTSD, and of PTSD’s effects on social cognition in BPD when the disorders co-occur.

### Dissociation in BPD, PTSD, and cPTSD

Dissociative symptoms are a cardinal feature of the BPD diagnosis, but as transient and not chronic states that occur “during periods of extreme stress … most frequently in response to real or imagined abandonment” (American Psychiatric Association, 2013, p. 664). Yet, a high proportion of individuals with BPD report clinically significant dissociative symptoms [7, 169]. Potential neurobiological substrates for dissociation in BPD have been identified, including increased glutamate concentrations in the ACC and impulsivity [170], and increased volume of the precuneus—the component of the self-referential DMN specifically implicated in episodic self-relevant memory [171].

The role played by PTSD or childhood traumatic victimization in dissociation in BPD has not been definitely established. Although limited in generalizability due to originating in a research site specializing in treatment of dissociative identity disorder (DID), a study of adult psychiatric inpatients predominantly (72%) diagnosed with BPD with a large sub-group diagnosed with DID (46%) conducted a detailed assessment of retrospectively-recalled childhood sexual and physical abuse [172]. Most (82%) of the DID-diagnosed patients met criteria for BPD, but about half of the BPD-diagnosed patients were not diagnosed with DID. Most of the inpatients (80%) had childhood exposure to sexual and/or physical abuse, but this was true of all of the comorbid BPD-DID patients and their trauma severity scores (including number of types, perpetrators, and duration of abuse) were 50% (vs. DID only) to 100% (vs. BPD only or other psychiatric disorders) higher than other patients’. The findings suggest that there may be a BPD sub-type characterized by severe dissociative symptoms, and by correspondingly severe childhood traumatic victimization.

Dissociation was included as a core feature in early formulations of cPTSD [173, 174], but its inclusion as a core feature of cPTSD has not been empirically supported [104]. There is psychobiological evidence supporting the existence of a dissociative sub-type of PTSD [30, 119, 175], particularly among individuals with histories of childhood traumatic victimization [176]. Moreover, dissociative disorders are common comorbidities of PTSD among individuals with histories of childhood traumatic victimization [177, 178].

Dissociation thus occurs in a sub-set of individuals with either BPD or PTSD/cPTSD, but whether these sub-groups overlap or are distinct has yet to be investigated. Dissociation could be a common factor linking BPD and cPTSD, or a sub-group with comorbid BPD and cPTSD, but it also could be a comorbidity associated with but independent of both BPD and cPTSD. Post-traumatic dissociation has been conceptualized as an extreme expression or endpoint of affect dysregulation [179], and therefore it may be affect dysregulation more generally rather than dissociation *per se* that connects (or differentiates) BPD and cPTSD.

### Affect dysregulation in cPTSD and BPD

Affect dysregulation specific to interpersonal stressors, actual or perceived, is a hallmark of BPD. Affect dysregulation in BPD phenomenologically and clinically involves high sensitivity to emotion in social contexts, heightened and labile negative affect, and both a deficit in adaptive and surfeit of maladaptive emotion regulation strategies [180]. Affect dysregulation (specifically, distress intolerance and deficits in adaptive emotion regulation and emotion clarity) were shown to account for the shame-based [181] heightened fear of social rejection in adults diagnosed with BPD [51, 52]. Problems with distrust and interpersonal lability and aggression in BPD have been linked to perceptual biases and cognitive deficits related specifically to a tendency toward perceived social exclusion/abandonment [163].

Functionally, affect dysregulation can be divided into under-regulation—difficulties in modulating and recovering from uncontrolled experiencing and expression of extremely intense forms of negative affect (e.g., rage, despair, impulsivity), and over-regulation (i.e., numbing, suppression, or dissociation) of positive and negative affect [158, 159]. Both over- and under-regulation of affect have been shown to be associated with BPD symptom severity [182], and under-regulation of affect has been shown to be associated with BPD diagnoses as well as to account for the relationship of childhood maltreatment history and chronic negative affect with BPD [183].

However, affect dysregulation is a factor in most if not all psychiatric disorders and not just BPD. For example, BPD diagnosed adults did not evidence a distinct pattern of physiological emotional processing – despite being physiologically activated in response to interpersonal challenge scripts – compared to those diagnosed with obsessive-compulsive disorder [184]. Moreover, affect dysregulation is heterogeneous in its expression within BPD. Cocaine-dependent BDP-diagnosed men, but not women, showed a greater tendency than cocaine-dependent men not diagnosed with BPD to focus their attention on cocaine-related stimuli after being exposed to trauma-related laboratory cues [185]. The researchers suggested that this may reflect an attempt by men who have comorbid BPD and cocaine-dependence to attempt to reduce emotional distress related to reminders of traumatic experiences. Although BPD-diagnosed women in a clinical sample had more affect regulation problems in general than non-BPD controls, a sub-group with comorbid avoidant personality disorder was found to have particularly severe deficits in tolerating distress and accessing adaptive emotion regulation strategies cognitively and physiologically (i.e., reduced heart rate variability when exposed to a stressor [186]. These findings raise the possibility that affect dysregulation—particularly under-regulation of affect due to impaired adaptive strategies and high levels of distress intolerance–may be a marker for a sub-group among BPD-diagnosed persons distinct from the majority who also struggle with affect dysregulation but tend to be tonically tolerant of distress and intermittently overwhelmed. The deficits in adaptive emotion regulation and problems with impulsivity and avoidance/self-medication characterizing this putative sub-group are consistent with descriptions of cPTSD.

Affect dysregulation thus may constitute a developmental pathway from childhood maltreatment to BPD. Latency/pre-adolescent children with BPD symptoms were found to be characterized by both chronic negative affect and impulsivity/disinhibition, with emotion-regulation deficits partially mediating those relationships [187]. Pre/early adolescent children with histories of emotional abuse were found to be at risk for developing BPD symptoms only if they also had problems with under-regulated affect and impulsivity [188]. A study with non-clinical adolescents found that distress intolerance, deficits in adaptive emotion regulation, and impulsivity each was independently associated with BPD symptoms [189].

However, affect dysregulation has been shown to play a major role in most Axis I and Axis II psychiatric disorders [190, 191]. Therefore, the specificity of affect dysregulation, and its precise nature, in BPD and cPTSD was investigated in a study of adult psychiatric inpatients who met criteria for BPD only, BPD with a comorbid somatoform disorder, or a somatoform or other severe Axis I disorder without BPD. Individuals with BPD were more likely than the other patients to have problems with under-regulation of affect (67% vs. 25% of somatoform disorder patients, most of whom also had problems with over-regulation), but one in three BPD participants did *not* have problems with under-regulation, including 20% who only had over-regulation problems [158, 159]. The most common profile for BPD patients (40%), with or without comorbid Axis I somatoform or other psychiatric disorders, was a combination of under- and over-regulation of affect. A similarly sizable (i.e., 40-50%) sub-group of individuals with BPD also reported clinically significant dissociation, almost always in combination with affect dysregulation [158, 159]. The combined dissociative/dysregulated presentation took three forms, either an excitatory state consistent with PTSD (e.g., trauma memory flashbacks), an inhibitory loss of awareness consistent with dissociative disorders and the dissociative PTSD sub-type (e.g., derealization, depersonalization), or the combined excitatory/under-regulated and inhibitory/over-regulated presentation. Over-regulation of affect was more prominent in somatoform disorder than BPD or other Axis I psychiatric disorders [158, 159], consistent with evidence linking somatoform disorders with alexithymia [192].

cPTSD was assessed in that psychiatric inpatient study as well [35, 36]. While most often comorbid with BPD (33%), cPTSD was *not* present in two of three BPD cases, and occurred most often when BPD and somatoform disorders were comorbid (38%). Thus, the combination of positive dissociation with under-regulated affect or negative dissociation with over-regulated affect was likely to best characterize cPTSD – but not BPD *per se*. BPD also was found to be associated with difficulty in recognizing emotions and distinguishing self-referential beliefs from reality, while anxiety and affective disorders were associated with difficulty in experiencing emotions and somatoform disorders were characterized by a deficit in self-referential beliefs.

These findings suggest that not only are affect dysregulation and dissociation complexly interrelated for different persons with BPD but that, based on clinical phenomenology, cPTSD is likely to extend beyond BPD. cPTSD includes features that reflect both under- and over-regulation of affect, positive and negative dissociation, difficulties in both experiencing and recognizing emotions, and both a surfeit and deficit in self-referential beliefs. A sub-set of BPD cases may, for example, represent a sub-type of cPTSD rather than vice versa. In such cases, a combination of traumatic victimization and disrupted primary caregiver attachment relationships might be expected, and this represents a final relevant line of research to review.

### The role of maltreatment by primary caregivers in BPD

Primary caregivers play an essential role in children’s development of emotion regulation capacities, and have been hypothesized to contribute to the risk of BPD when they fail to bond and attune emotionally with their child [83] or to protect their child from victimization [193]. However, most children whose primary caregivers are dysregulated or insufficiently protective—or even abusive—do not develop BPD [194]. Maltreatment by primary caregivers is, however, consistently associated with children’s emotion dysregulation [195, 196]. Therefore, we next consider the clinical and research evidence linking primary caregiver relationships, and specifically maltreatment in those relationships, with BPD.

Emotion regulation is known to be founded on developmental attainments (e.g., emotion self-awareness, empathy, self-control) that are profoundly influenced by interaction with primary caregivers beginning in infancy and extending through childhood [197]. Affect dysregulation in adolescence and adulthood correspondingly has been found to be associated with childhood relationships with primary caregivers who are unresponsive, poorly attuned, or punitive [90]. Consistent with this, individuals with BPD often report childhood histories of primary caregiver neglect, abuse, invalidation, or impairment [79, 83, 198]. In a combined concurrent and retrospective study, young women meeting BPD criteria were particularly likely compared to age-matched women with no psychiatric diagnosis to report feeling unprotected in their relationship with their mother and to have elevated cortisol levels at the outset of a laboratory interaction with their mother [193]. Infants of mothers with BPD also were found to be less attentive and contact-seeking with their mothers, who in turn were found to be less responsive, able to provide structure, satisfied and self-confident [199], and more likely to express fear or disorientation [200] in their interactions with their infant children, compared to mothers with no psychiatric diagnosis.

Although compromised childhood attachment relationships with primary caregivers may be a key factor in the development of BPD, most children with insecure or disorganized attachment relationships and most adults who recall a troubled, insufficiently protective, or even frankly rejecting or severely neglectful relationship with primary caregivers do not develop BPD [81]. It is possible that only explicitly traumatic experiences in relation to primary caregivers account for vulnerability to or the etiology of BPD—in which case the sub-group of individuals with BPD who have experienced traumatic victimization by (or in relation to) a primary caregiver may be better characterized by cPTSD either as a BPD comorbidity or as the primary disorder. Pursuing that question, a study compared psychiatric inpatients diagnosed with BPD to those with severe somatoform or mood/anxiety disorders and found that the BPD cases were most likely to report traumatic victimization by a primary caregiver and severe under-regulation of affect [35, 36]. Moreover, under-regulation of affect partially mediated the relationship between traumatic victimization by a primary caregiver and BPD [201]. A history of traumatic victimization by primary caregiver was reported by most BPD-diagnosed inpatients (75%), but also was reported by a majority (51%) of the inpatients with Axis I disorders [35, 36].

Consistent with most approaches to psychotherapy for BPD [202], trusted and trustworthy relationships can serve a protective role, reducing the risk of developing BPD characteristics when a child experiences traumatic victimization in primary caregiving relationships. A study with college students found that having valued relationships outside the family in the larger community statistically mediated—and mitigated against—the risk of developing BPD traits associated with a history of exposure to childhood betrayal trauma [203]. These findings suggest that the role of traumatic victimization in the etiology of BPD may be obscured in many fortunate cases where protective relationships mitigate against its adverse effects.

### Implications for clinical practice and research

Findings from this review of clinical and neurobiological research suggest that BPD may involve heterogeneity related to psychological trauma that includes but extends beyond comorbidity with PTSD and potentially involves childhood victimization-related dissociation and affect dysregulation consistent with cPTSD. BPD and cPTSD overlap substantially, but it does not seem warranted to conceptualize cPTSD either as a replacement for BPD nor simply as a sub-type of BPD. Persons with severe childhood traumatic victimization histories are at risk for BPD, PTSD, and cPTSD, but the clinical phenomenology and neurobiology of the three syndromes are distinct: unlike severe PTSD or cPTSD, BPD does not always involve traumatic antecedents but usually involves severe attachment insecurity and disorganization. The implications and unanswered questions raised by these findings will next be discussed.

### Assessment

Clinicians evaluating severely distressed clients with significant trauma histories may find that clearly distinguishing BPD and cPTSD can increase their precision in assessing and formulating treatment plans to address complex forms of affect dysregulation and dissociation which often accompany the trauma-related hypervigilance and dissociative features of PTSD. When abandonment terror is combined with habituation to extreme distress and alternating idealization and devaluation of self and others are prominent, as well as a history of neglect or otherwise compromised childhood relationships with primary caregivers, the careful safety planning and affect identification skills that are hallmarks of efficacious BPD treatment are indicated [202]. On the other hand, when hypervigilance and intrusive re-experiencing centers on fear of trusting self (including one’s own bodily reactions, emotions, and thoughts) or others (who are perceived as unreliable but not fundamentally devalued) to recognize and handle threats, and there is a history of abuse or emotional betrayal in primary relationships, the affect modulation and self-reparative skills that are central to efficacious cPTSD treatment are indicated [204, 205]. If only PTSD is assessed, for example with a severely and chronically poly-victimized client, the evaluation may fail to identify crucial components of affect dysregulation and altered relational- and self-schemas that may shape, amplify or perpetuate intrusive re-experiencing, avoidance, pervasive negative affect states and beliefs, hypervigilance, and dissociation. Although, as noted above, the *DSM-5* PTSD criteria have been expanded to include aspects of affect, relational, and self-dysregulation, the emphasis in PTSD remains upon re-experiencing and hypervigilance with dysregulation not a fully delineated as in cPTSD and BPD.

Considered together within a cPTSD framework, PTSD and BPD could provide a phenomenological and nosological understanding of the separate and combined impacts of the survival threat central to PTSD [206] and the invalidation of self that is a core feature of BPD [207]. A cPTSD integration yields a complex formulation that is more than the sum of its parts [208]. For example, a cPTSD perspective could clarify how a client with a history of betrayal trauma [62] may experience dissociative episodes alternating with rage and impulsive/reckless behavior. In such a case, if relational stressors elicit extreme distress or alternating devaluing and idealization in current relationships related to perceived or actual abandonment, violation, and shaming, BPD would be a focal concern. If avoidance of perceived or actual threat of suffering or causing harm in current relationships (along with dysphoria, dissociation, and/or hyperarousal) was debilitating, PTSD would be focal. However, if assessment indicated the presence of affectively charged (or incongruously emotionally numbed) schemas representing self and relationships as simultaneously irreparably damaged but essential and irreplaceable, and this conflict was associated with affective over- as well as under-regulation, cPTSD offers a unique focus distinct from that of either BPD or PTSD. This formulation could demystify complex pathological adaptations for the client and clinician, drawing on mechanisms known to underlie PTSD and BPD without attempting to artificially graft the two of them together (along with other comorbid diagnoses) and unnecessarily increasing the cost and burden due to multiply layered treatment, as well the risk of treatment failure and stigma [10].

### Treatment

Advances in empirically-supported treatment for both PTSD and BPD illustrate how an integrative cPTSD framework can yield therapeutic advances. Dialectical Behavior Therapy (DBT) is well-established based on efficacy as well as effectiveness research and widespread acceptance by practicing clinicians, yet its primary benefits over and above community treatment by expert clinicians have been shown to be preventing self-harm and enhancing interpersonal functioning by reducing experiential avoidance and expressed anger [209]. These are critically important outcomes, but DBT did not show greater effectiveness than expert treatment as usual (TAU) with regard to guilt, shame, anger suppression, anxiety, core schemas, and impulse control—*symptoms that are central to cPTSD*. Improvements occurred for recipients of both DBT and TAU, but the utility of those findings is limited by the fact that “expert” treatment cannot easily by deconstructed and taught to other clinicians—and generations of expert therapists have had limited success altering the course of BPD. An intriguing possibility suggested by our review, therefore, but as yet untested, is that clinicians who have become deeply knowledgeable about the challenges of treating individuals with BPD have developed intuitive strategies for helping their clients to understand and gradually gain a sense of mastery of the pervasive distrust and hypervigilance toward their own and other persons’ emotions—viewing these as threats—that are common sequelae of traumatic abuse and betrayal and subsequent cPTSD.

Recognizing that traumatic stress reactions may need to be addressed deliberately for many individuals diagnosed with BPD, an adaptation of DBT has been developed with a modified form of Prolonged Exposure (PE) therapy for PTSD. When pilot tested with women meeting criteria for comorbid BPD and PTSD, the trauma-informed DBT yielded similar completion rates (67-70% when therapists had acceptable fidelity) and better outcomes for self-harm, depression, anxiety, guilt, and shame than DBT alone [210]. While not referring explicitly to cPTSD, the trauma-informed DBT intervention not only addressed PTSD with the standard features of PE, but specifically modified PE to:

1. monitor potential negative reactions to exposure (e.g., pre-post exposure ratings of urges to commit suicide and self-injure), (2) target problems that may occur during or as a result of exposure (e.g., dissociation, increased suicide urges), and (3) utilize therapist strategies (e.g., dialectics, irreverence, self-disclosure, validation) that address the particular characteristics of severe BPD patients. In addition, structured procedures for managing common complexities … during PTSD treatment with this population were used, including strategies to: (1) address multiple traumas, including experiences that do not meet the *DSM*-IV definition of trauma (e.g., severe verbal abuse), (2) conduct imaginal exposure with fragmented trauma memories, and (3) target unjustified trauma-related shame. In addition, the DBT PE protocol includes a requirement that the protocol be stopped (ideally temporarily) if any form of intentional self-injury recurs.

In other words, by considering the interactive effects of exposure to multiple types and forms of both traumatic victimization and invalidating relationships, the PTSD treatment component was adapted to support the affect, self, and relational regulation necessary to regain trust in self and in sustaining relationships – precisely the goal and focus of cPTSD treatments.

A recent randomized controlled trial study tested the efficacy of a similar combination of trauma processing therapy and DBT (DBT-PTSD) with 74 women diagnosed with PTSD related to childhood sexual abuse [211]. Half of the participants met criteria for comorbid BPD. DBT-PTSD was associated with substantial reductions in PTSD severity for women with or without BPD, while those in a wait-list treatment-as-usual condition showed almost no change (i.e., reductions on average of 33 vs. 2 points on the Clinician Administered PTSD Scale). Dysfunctional behaviors such as self-harm were monitored carefully and did not increase for participants in DBT-PTSD. These findings provide an independent and well-controlled replication demonstrating the efficacy of combining trauma narrative processing with DBT for women with CSA-related PTSD and BPD. Whether the women also met criteria for cPTSD was not assessed and represents a key question for further studies of DBT-PTSD.

The potential for bidirectional synergistic enhancement of treatment for both PTSD and BPD shown by this adaptation of DBT is further illustrated by adaptations to evidence-based therapies for PTSD with clients who are not diagnosed with BPD but have complex trauma histories and cPTSD symptoms and impairments. Structured Therapy for Affective and Interpersonal Regulation with Modified Prolonged Exposure (STAIR-MPE) provides a DBT-informed first phase of therapy aimed at enhancing affect and interpersonal regulation skills, followed by a modification of PE similar to that in DBT+PE which carefully titrates trauma memory processing intensity to not exceed the client’s affect regulation capabilities. Two randomized controlled trial studies have demonstrated STAIR-MPE’s efficacy in reducing severe PTSD, depression, and dissociation with women with chronic childhood victimization- or interpersonal violence-related PTSD [212–214]. Trauma Affect Regulation: Guide for Education and Therapy (TARGET) is an alternative DBT-informed cPTSD therapy, which engages clients in trauma processing with a present-centered cognitive therapy rather than exposure or other trauma memory-focused techniques. TARGET teaches a sequential set of arousal modulation, self-referential focusing, and affect regulation skills designed to reduce avoidance and increase awareness and intentionality in relation to trauma-related internal states in order to address the postulated mechanisms of flashbacks and intrusive memories. In three randomized controlled trial effectiveness studies with adults with cPTSD [13, 14, 215, 216] and one with adolescents [217], TARGET has achieved reductions in PTSD, depression, affect dysregulation, substance use risk, and alienation superior to those of active comparison therapies. Thus, a cPTSD framework informed by BPD clinical phenomenology and therapeutic approaches has resulted in promising innovations that can make therapy more effective and accessible for trauma survivors whose affect, self, and interpersonal dysregulation otherwise may bar them from, or lead them to refuse, PTSD treatment [11, 12].

### Research

At a foundational level, the scientific challenge is to develop and test a nomologically- systematic and empirically-grounded theory of interrelationships of: (1) biosocial *diatheses* (e.g., genetic/familial/ neurobiological risk /protective factors), (2) attachment/relational (e.g., absent or poorly attuned caregiving) and more frankly traumatic (e.g., physical or sexual abuse) *stressors* separately and in varied combinations (e.g., caregiver-perpetrated abuse or attachment-disrupting family violence), and (3) *developmental trajectories* (e.g., biopsychosocial adaptations leading to static or changing functioning) in the domains of impairment, resilience, or optimal growth and functioning, in order to disentangle the associations among BPD, PTSD, and cPTSD.

More specifically, a systematic comparison of how the three syndromes differ on the National Institute of Mental Health’s Research Domain Criteria (RDOCs) [218] could clarify the syndromes’ construct validity (or lack thereof) and point toward clinical and translational studies of targeted assessment, diagnostic, and therapeutic protocols. For example, in the RDOC “negative valence systems” PTSD should be particularly associated with acute or sustained threat and BPD with frustrative nonreward, while the two would be expected to overlap on the criteria of anxiety and loss, across the range of units of analysis from genes to physiology to behavior and self-report. cPTSD would be theoretically and clinically supported if there was evidence of unique profile(s) across the criteria and units of analysis distinct from both BPD and PTSD (e.g., specific genetic and familial vulnerability to anxiety and frustrative nonreward on the physiological, behavioral, and psychological level following sustained threat and loss). The accuracy of identification of distinct profiles for BPD, PTSD, and cPTSD is likely to increase dramatically when other RDOC domains (i.e., positive valence and arousal/ regulatory systems, cognitive and social processes) are included in characterizing the adaptations made by sub-groups with different diathesis-stressor histories. BPD’s sensitivity to abandonment and rejection, PTSD’s arousal and positive affect dysregulation, and cPTSD’s altered declarative memory, hypo-arousal, and self-perception are potential foci for a more differentiated description of core impairments that could justify distinct classifications and better tailored treatment strategiess.

Empirically identified targets for assessment and diagnosis are critical to refined therapeutic interventions for BPD, PTSD, and cPTSD. For example, comorbid BPD+PTSD may be responsive to the distress tolerance and avoidance-reduction therapies which have strong evidence bases for those disorders. However, the high drop-out rate reported even by potentially efficacious combined BPD+PTSD interventions [210] may signal the need for alternative therapeutic frameworks such as those offered by cPTSD therapies that do not modify exposure-based interventions but instead directly address dissociation [213] or severe affect dysregulation [215]. The SMART adaptive treatment research paradigm provides an efficient approach to matching non-responders to alternative therapeutic approaches based on characteristics (e.g., RDOC criteria) that are not targeted by first line evidence-based treatments but commonly present among non-responders [219]. Rather than fitting recipients to the therapy, this approach to treatment research matches therapies to recipient characteristics when this is necessary to reduce the number needed to treat for successful outcomes. RDOC-like profiles of BPD, PTSD, and cPTSD could provide a rational and efficient basis for SMART treatment outcome research, as well as for refinements in the specific processes and interventions used by each therapy in order to address key features of specific syndromes.

## Conclusion

This review has summarized current clinical and scientific findings regarding comorbidity, clinical phenomenology and neurobiology of BPD, PTSD, and cPTSD, The role of traumatic victimization in general and relational/attachment trauma specific to primary caregivers, dissociation, and affect dysregulation were highlighted. The etiology and course of BPD involves heterogeneity related to psychological trauma that includes, but extends beyond, comorbidity with PTSD and potentially involves childhood victimization-related affect dysregulation and dissociation consistent with a cPTSD framework. Although BPD and cPTSD overlap substantially (as do cPTSD and PTSD), it is unwarranted to conceptualize cPTSD either as a replacement for or sub-type of BPD. The evidence instead suggests that a sub-group of BPD patients—who often but not always have comorbid PTSD—may be best understood and treated if cPTSD is explicitly addressed as well—and in some cases, in lieu of—BPD. A better differentiated empirically-grounded view of cPTSD, BPD, and PTSD is a high priority for the advancement of clinical practice and research with traumatized adults.
